# Designing Personalized Antigen-Specific Immunotherapies for Autoimmune Diseases—The Case for Using Ignored Target Cell Antigen Determinants

**DOI:** 10.3390/cells11071081

**Published:** 2022-03-23

**Authors:** Jide Tian, Min Song, Daniel L. Kaufman

**Affiliations:** Department of Molecular and Medical Pharmacology, UCLA School of Medicine, University of California, Los Angeles, CA 90095-1735, USA; minsong1961@hotmail.com

**Keywords:** antigen-specific immunotherapy, type 1 diabetes (T1D), autoimmune disease, regulatory T cells, precursor T cell, preproinsulin, insulin, GABA, NOD mice, intervention, naive T cell, newly diabetic, antigen-specific therapy, antigen-based therapy, personalized medicine, precision medicine, Treg, Tr1

## Abstract

We have proposed that antigen-specific immunotherapies (ASIs) for autoimmune diseases could be enhanced by administering target cell antigen epitopes (determinants) that are immunogenic but ignored by autoreactive T cells because these determinants may have large pools of naïve cognate T cells available for priming towards regulatory responses. Here, we identified an immunogenic preproinsulin determinant (PPI_L4-20_) that was ignored by autoimmune responses in type 1 diabetes (T1D)-prone NOD mice. The size of the PPI_L4-20_-specific splenic naive T cell pool gradually increased from 2–12 weeks in age and remained stable thereafter, while that of the major target determinant insulin B-chain_9-23_ decreased greatly after 12 weeks in age, presumably due to recruitment into the autoimmune response. In 15–16 week old mice, insulin B-chain_9-23_/alum immunization induced modest-low level of splenic T cell IL-10 and IL-4 responses, little or no spreading of these responses, and boosted IFNγ responses to itself and other autoantigens. In contrast, PPI_L4-20_/alum treatment induced robust IL-10 and IL-4 responses, which spread to other autoantigens and increased the frequency of splenic IL-10-secreting Treg and Tr-1-like cells, without boosting IFNγ responses to ß-cell autoantigens. In newly diabetic NOD mice, PPI_L4-20_, but not insulin B-chain_9-23_ administered intraperitoneally (with alum) or intradermally (as soluble antigen) supplemented with oral GABA induced long-term disease remission. We discuss the potential of personalized ASIs that are based on an individual’s naïve autoantigen-reactive T cell pools and the use of HLA-appropriate ignored autoantigen determinants to safely enhance the efficacy of ASIs.

## 1. Introduction

Antigen-specific immunotherapy (ASI) for autoimmune disease is a theoretically appealing treatment strategy because it may induce regulatory T cell responses to target tissue antigens with little interference with immune system function (reviewed in [1,2,3]). The results from clinical trials with individuals newly diagnosed with T1D who were given different ß-cell autoantigens as monotherapies have supported the safety of ASIs and their capacity to induce shifts in the balance of immune responses to ß-cell autoantigens that are expected to be beneficial. Yet, clinically relevant improvements are elusive [2,3], although an analysis of the combined data from several clinical trials suggests that GAD65/alum monotherapy had some ability to reduce the loss of C-peptide production [4]. Currently, there are a number of ongoing clinical trials that are testing ASIs in combination with other treatments on individuals newly diagnosed with T1D. The results from a phase II clinical trial that tested intra-lymphatic GAD65/alum administration along with vitamin D supplementation hold promise that this treatment may slow the loss of residual insulin production after T1D onset in an HLA-restricted subset of new-onset T1D patients [5,6].

Further enhancements of ASI’s ability to help inhibit the loss of insulin production in newly diabetic individuals are sorely needed. However, ASI development is hampered by a lack of guiding principles as to which target tissue antigens should be selected for ASI trials and the best route ASI of administration. These issues have been difficult to assess in clinical trials since it would require many different treatment arms and there are limited numbers of new-onset T1D patients willing to enroll in clinical trials. There is also little information from the widely used NOD mouse model of spontaneous T1D on how to optimally design interventive ASIs because ASI monotherapies are ineffective in this model after T1D onset.

Traditionally, ASIs have focused on treating with whole autoantigens, peptides containing major target epitopes (or “determinants”) of autoreactive T cells, or peptides eluted from MHC, with the goal of inducing regulatory T cell responses. These ASI strategies lead to the increased presentation of autoantigen determinants that are often already targets of activated autoimmune responses such that (1) there may be few naïve cognate T cells available for priming by ASIs due to their recruitment into the pathogenic autoimmune response, and (2) there is a danger of boosting existing cognate effector T cells. Notably, basic immunological studies of antigen immunogenicity have shown that it is the frequency of cognate naïve T cell precursors that determines the magnitude of the T cell response to an immunogen [7,8,9]. Accordingly, we have hypothesized that the antigens used for ASIs should not be chosen based on the frequency of activated proinflammatory cognate T cells (i.e., being a major target determinant), but rather the size of the naïve T cell precursor pool that is available for priming toward regulatory responses. The use of immunogenic target cell autoantigen determinants which are ignored by the autoimmune response, i.e., ignored determinants (IDs), would be the converse of traditional ASI approaches. Yet, this approach has parallels recent advances in cancer treatment that are based on vaccination with tumor neoantigens which are highly immunogenic because they bypassed central tolerance mechanisms and can induce more robust anti-tumor T cell responses in cancer patients (e.g., [10,11]).

To begin to test this therapeutic approach, we previously studied the immunogenicity of different ß-cell and non-ß-cell antigens to prime IL-4-secreting T cell responses in 6 and 12 weeks old prediabetic NOD mice. We observed that the ability of foreign and non-ß-cell self-antigens to prime T cell IL-4 responses did not diminish with disease progression in NOD mice. In contrast, the ability of whole ß-cell autoantigens, or synthetic peptides containing major autoantigen target determinants, to prime IL-4 secreting T cell responses is greatly attenuated with disease progression [9,12]. We hypothesized that this attenuation was due to the recruitment of naïve autoantigen-reactive T cells into the autoimmune response, leaving fewer available for priming by ASI towards regulatory responses [9]. To circumvent this, we identified immunogenic ß-cell autoantigen IDs. We observed that, unlike target determinants, the ability of these IDs to prime regulatory responses was unaffected by disease progression and that prophylactic immunization of prediabetic NOD mice at 12 weeks in age with the IDs conferred significantly better protection from developing T1D than ASI using peptides containing target determinants [9]. The immunogenicity of the determinants, and the extent of spreading of IL-4 responses to other ß-cell autoantigens, correlated with the efficacy of ASIs to inhibit subsequent T1D development [9]. Supporting these findings, a study of dendritic cell-based immunotherapy in which NOD mouse dendritic cells were loaded with an immunodominant, subdominant, or ignored ß-cell autoantigen determinant, observed that dendritic cells loaded the latter two types of ß-cell antigen-induced more Tregs and appeared to be more efficacious therapeutics [13].

Here, we identified a new immunogenic ID within mouse preproinsulin’s leader sequence (PPI_L4-20_) that appears not to be targeted by spontaneous autoimmune responses in NOD mice. Second, using peptide/MHC tetramers, we monitored the size of the naïve PPI_L4-20_-specific T cell pool, as well as that of the widely studied major target determinants within insulin B-chain_9-23_ (hereafter “insulin B_9-23_”) over the course of NOD mouse development. We also assessed the effects of immunizing 15–16 weeks old NOD mice with these antigens on their IL-10, IL-4, and IFNγ-secreting T cell responses to the immunogen and other ß-cell autoantigens, as well as the frequency of splenic CD4^+^FoxP3^+^ Tregs and CD4^+^Foxp3^-^IL-10^+^ Tr1-like cells. Finally, we report on the efficacy of ASIs based on intraperitoneal administration (with alum) vs. intradermal delivery of low doses of soluble antigen in combination with low-dose oral GABA treatment to induce long-term disease remission in newly diabetic NOD mice. The results suggest that the naïve cognate CD4^+^ T pool size in an individual may provide a biomarker of an ASI’s ability to prime regulatory responses and hence, its effectiveness. Furthermore, personalized ASIs based on the availability of naïve T cells, particularly naïve ID-reactive T cells, may provide safer and more effective ASIs for treating autoimmune diseases.

## 2. Materials and Methods

### 2.1. Mice

NOD mice (Taconic Farms, Germantown) were bred and maintained under specific pathogen-free conditions with a 12-h light/dark cycle in the Division of Laboratory Animal Medicine at UCLA. They were provided food and water ad libitum. This study was carried out according to the Guide for the Care and Use of Laboratory Animals of the National Institutes of Health. The protocols for all experiments using vertebrate animals were approved by the Animal Research Committee at UCLA (protocol #1993–2001).

### 2.2. Antigens

We examined the mouse preproinsulin I protein for regions with a consensus I-A^g7^ binding site [14,15,16], but did not span a region already known to be a target of autoreactive T cells in NOD mice. We synthesized a 17-mer peptide containing preproinsulin I leader sequence amino acids 4–20 (PPI_L4-20_, LVHFLPLLALLALWEPK) and a peptide containing preproinsulin amino acids 82–93 (PPI_82-93_, VARQKRGIVDQC) at >98% purity (JPT, Germany). Peptides containing the major ß-cell autoantigen target determinants mouse insulin B_9-23_ [17,18,19,20], GAD_524-543_ (also termed GADp35 [20], and heat shock protein-peptide 65 (HSP p277 [21]), as well as an immunogenic control peptide from hen egg lysozyme (HEL_11-25_, AMKRHGLDNYRGYSL), were synthesized at >98 purity (GenScript). Insulin B-chain and human proinsulin were purchased from Sigma-Aldrich and AmideBio, respectively.

### 2.3. T Cell Proliferation Assay

To determine the immunogenicity of synthesized peptides, NOD mice at 6–8 weeks of age were immunized with the indicated peptide (50 µg each) in 50% complete Freund’s adjuvant (CFA) in their footpads, a method that is thought to induce maximal proinflammatory T cell responses [22]. Nine days later, their draining lymph nodes were dissected, and lymph node mononuclear cells were isolated. Lymph node mononuclear cells (4 × 10^5^ cells/well) were stimulated in triplicate with each peptide at 7–20 µM in HL-1 medium in 96-well plates for 72 hr. Anti-CD3 (1 µg/mL, Biolegend) and purified protein derivative (5 µg/mL) were used as positive controls while the cells, in the absence of any antigen challenge, served as a negative control. During the last 16-h of incubation, ^3^H-thymidine (1 µCi/well) was added, and ^3^H-thymidine uptake in individual wells was measured by a β-counter.

### 2.4. ELISPOT Analysis of the Numbers of Spontaneously Arising Splenic and Pancreatic Infiltrating IFNγ-Secreting Responses to Antigens

To test for spontaneous T cell responses to β-cell antigens, splenic mononuclear cells were isolated from unmanipulated female NOD mice at 12–13 weeks of age, at which time they display strong splenic Th1 responses to many β-antigens [20]. Splenic mononuclear cells (10^6^/well), or a mixture of pancreatic lymph node and islet-infiltrating mononuclear cells (10^5^/well), were tested for their IFNγ responses to proinsulin insulin B chain, insulin B_9-23_, and PPL_L4-20_ by an ELISPOT assay which is detailed below.

### 2.5. MHCII Tetramers and FACS Analysis

Phycoerythrin (PE)-labeled I-A^g7^ mouse tetramers loaded with PPI_L4-20_, the high-affinity insulin B_9-23_ mimotope InsBp8E (HLVERLYLVCGEEG, [18]) or HEL_11-25_ peptides were prepared by the NIH Tetramer Core Facility. Splenic mononuclear cells were isolated from female NOD mice at the indicated age and counted. The cells were pre-treated with anti-CD16/anti-CD32 to block FcR and CD4^+^ T cells were purified by negative selection based on nanobead-based magnetic separation using the Mojosort mouse CD4 T cell isolation kit (Biolegend), according to the manufacturer’s instructions. Briefly, the mononuclear cells were stained with a biotinylated antibody cocktail. The labeled cells were reacted with streptavidin nanobeads and bound by magnetic isolation. The unbound CD4^+^ T cells were collected and counted. Subsequently, the isolated CD4^+^ T cells (1 × 10^6^ cells/tube) were stained with FITC-anti-CD4 and APC-anti-CD25 as well as each tetramer (1 µg each) on ice for 30 min. After being washed with PBS, the frequency of CD4^+^CD25^-^PE^+^ naïve T cells were determined by flow cytometry in a BD LSII Flow cytometer using BD FACSDiva™ Software (BD Biosciences). Finally, the numbers of CD4+CD25-PE+ naïve T cells were calculated based on their percentages and the total number of CD4^+^ T cells isolated.

### 2.6. ELISPOT Analysis of Autoimmune Responses in Antigen-Immunized Mice

Female NOD mice at the indicated age were injected intraperitoneally (IP) with 100 μg of the indicated antigen peptide in 50% incomplete Freund’s adjuvant (IFA), an adjuvant that drives highly Th2-polarized immune responses [22]. Ten days later, the treatment was repeated. After another ten days, the frequency of antigen-specific splenic T cells secreting IL-10, IL-4, or IFNγ was determined using a modified ELISPOT technique as previously described [23,24]. Briefly, 10^6^ splenic or 5 × 10^5^ mixed pancreatic lymph node and islet infiltrating mononuclear cells were added per well (in duplicate) of an ELISPOT plate (Millipore) that had been coated with cytokine capture antibodies and incubated with the indicated synthetic peptide (20 µM), proinsulin (100 µg/mL), or insulin B-chain (100 µg/mL) for 24 hrs for IFNγ, or 40 hrs for IL-4 and IL-10 detection. After washing, biotinylated detection antibodies were added and the plates were incubated at 4 °C overnight. The bound secondary antibodies were visualized using horseradish peroxidase (HRP)-streptoavidin (DAKO) and 3-amino-9-ethylcarbazole. Antibodies XMG 1.2/ biotinylated R4-6A2, 11B11/Biotin-BVD6-24G2 and JES5-16E3/Biotin-JES5-2A5 (Biolegend) were used for capture and detection of IFNγ, IL-4, and IL-10, respectively.

### 2.7. Treatments

We monitored the blood glucose levels of female NOD mice beginning at 12 weeks of age and those which displayed blood glucose levels >250 mg/dL but <300 mg/dL on two consecutive days were considered as newly diabetic. At the time of disease onset, some mice were given IP the indicated antigen (100 µg) that was complexed to alum (Pierce, Rockford), a clinically applicable adjuvant which induces Th2-type immunity [22] and placed on water which contained GABA (Sigma-Aldrich, St. Louis, MO, USA) at 6 mg/mL. Our previous dose-response studies of GABA treatment in newly diabetic NOD mice showed that GABA at 6 mg/mL was the lowest dose with some ability to reverse hyperglycemia, although mice given this low-dose quickly became hyperglycemic again (within two weeks) [25]. Following immunization, the mice received GABA treatment continuously thereafter and were boosted with the same dose of peptide/alum 10 days after the initial immunization. For studies of intradermal antigen delivery, newly diabetic female NOD mice were immunized intradermally with one microgram of PPI_L4-20_ or Ins B_9-23_ in 100 µL of PBS at multiple skin sites once weekly for four consecutive weeks (as in [26]) and were given GABA continually through their drinking water (6 mg/mL). The drinking water was changed every five days. Previous studies have shown that mice given GABA through their drinking water consume a similar amount of food and water as mice on plain water [25,27]. Following treatments, their blood glucose was monitored three times per week. Individual mice with two consecutive blood glucose readings < 250 mg/dL were considered to be in remission, after which two consecutive blood glucose readings > 250 mg/dL were considered to be disease relapse. Mice with two blood glucose > 400 mg/dL were humanely euthanized.

### 2.8. Analysis of Treg and Tr-1-like Cell Frequencies

To analyze the frequency of splenic CD4^+^Foxp3^+^ Tregs and CD4^+^Foxp3^-^IL-10^+^ Tr1 cells, splenic mononuclear cells were isolated from the mice and treated with anti-CD16/anti-CD32. After being washed, the cells (1 × 10^6^/tube) were stained in duplicate with FITC-anti-CD4, fixed and permeabilized, and intracellularly stained with PE-anti-Foxp3 alone or PE-anti-Foxp3 and APC-anti-IL-10, followed by washing. Control cells were stained with isotype controls. The frequency of splenic CD4^+^Foxp3^+^ Tregs and CD4^+^Foxp3^-^IL-10^+^ Tr1-like cells was determined by flow cytometry. To quantify splenic CD4^+^Foxp3^+^ Tregs, the cells were gated on the control, and the frequency of CD4^+^ and CD4^+^Foxp3^+^ Tregs were analyzed. To quantify the frequency of CD4^+^Foxp3^-^IL-10^+^ Tr1-like cells, the cells were gated on the control and then on CD4^+^Foxp3^-^ cells. The frequency of CD4^+^Foxp3^-^IL-10^+^ Tr1-like cells was determined.

## 3. Results

### 3.1. Identification of an Immunogenic Ignored Determinant within Preproinsulin

**Figure 1:**
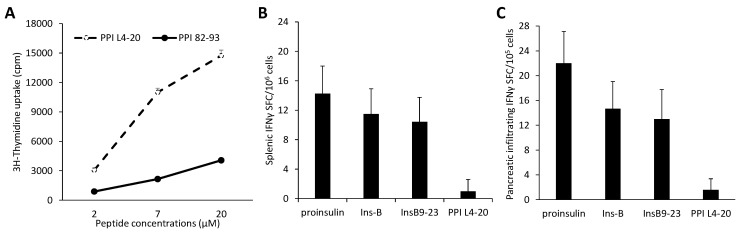
PPI_L4-20_ is an immunogenic preproinsulin determinant that is ignored by spontaneous autoimmune responses in NOD mice. (**A**) Lymph node T cell proliferation following immunization with PPL_L4-20_ and PPI_82-93_ and challenge with each immunogen. To determine whether PPL_L4-20_ and PPI_82-93_ contain immunogenic determinants of PPI, female NOD mice (6–8 weeks old) were immunized with PPL_L4-20_ and PPI_82-93_ (50 µg of each in 50% CFA) in their food-pads. Nine days later, the mononuclear cells from the draining lymph nodes of individual mice were stimulated with PPI_L4-20_ (dashed line) or PPI_82-93_ (solid line) at the indicated concentration for 72 h. The antigen-specific T cell proliferation was determined by ^3^H-thymidine uptake. Data are expressed as the mean ± SD of each group (*N* = 4) from two separate experiments. The control cells without antigen stimulation displayed mean counts from 679 to 834 cpm (data not shown) while the positive group of cells that had been stimulated with anti-CD3 (1 µg/mL) or PPD (5 µg/mL) displayed means of 23,540 or 18,760 cpm, respectively. (**B**,**C**) ELISPOT analysis of the numbers of spontaneously arising (**B**) splenic and (**C**) pancreatic infiltrating IFNγ-secreting T cell responses to ß-cell antigens. Splenic mononuclear cells or a mixture of pancreatic lymph node and islet-infiltrating mononuclear cells that were isolated from 12–13 weeks old unmanipulated female NOD mice were tested for their IFNγ responses to proinsulin, insulin B chain, insulin B_9-23_, and PPL_L4-20_ by ELISPOT assays. The data are expressed as the mean ± SD of spot forming cells (SFC) in 10^6^ splenocytes, or 10^5^ pancreatic infiltrates in each group (*n* = 6) from two separate experiments. The number of SFC in the control was ≤3 and the number of SFC from anti-CD3 stimulated cells was about 220.

We focused on mouse preproinsulin since it is a key target of autoimmune responses. We sought to identify immunogenic determinants within the mouse preproinsulin I protein that did not become targets of spontaneous autoimmune responses. Towards this, we synthesized two peptides, PPI_L4-20_ from the preproinsulin leader sequence and PPI_82-93_, which contain a consensus I-Ag^7^ binding sequence [14,15,16] but do not span regions already known to be targets of autoreactive T cells in NOD mice. Following immunization with each peptide in 50% CFA in the footpads of 6–8 weeks of NOD mice, we found that immunization with PPI_L4-20_, but not PPI_82-93,_ mounted strong lymph node T cell proliferation ex vivo (Figure 1A). These data indicate that the PPI_L4-20_ has potent immunogenicity and can induce T cell responses in NOD mice.

We next assessed whether PPI_L4-20_ was a target of spontaneous autoimmune responses in 12–13 weeks old NOD mice by testing their splenic mononuclear cells, as well as a combined mixture of pancreatic lymph node and islet-infiltrating T cells for IFNγ-secreting PPI_L4-20_-reactive T cells by an ELISPOT assay. We detected splenic (Figure 1B) and pancreatic lymph node/islet-infiltrating (Figure 1C) IFNγ-secreting T cells responding to proinsulin, insulin B-chain, and insulin B_9-23_, but not to PPI_L4-20_. Moreover, subsequent ELISPOT studies of splenic T cell responses in ~18–19 weeks old NOD mice also found no evidence of T cell responses to PPI_L4-20_ at this near diabetic stage (see below). These data indicate that PPI_L4-20_ is an immunogenic preproinsulin determinant that appears to be ignored by spontaneous autoimmune responses in NOD mice.

### 3.2. A Large Pool of Cognate L4-20-Reactive Naïve T Cells Is Available for Priming during Late-Stage Pre-T1D

**Figure 2:**
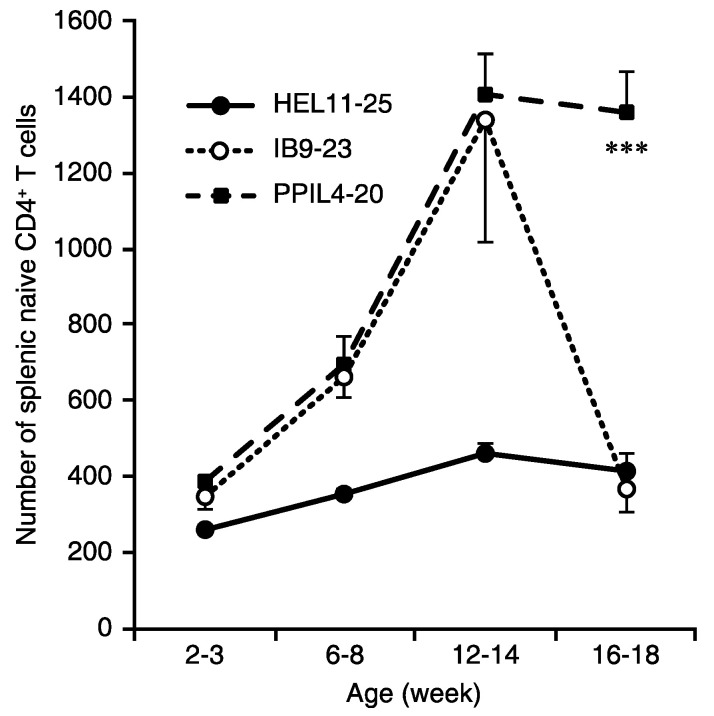
A large pool of naive CD4^+^ T cells recognizing PPI_L4-20_ is available in near-diabetic 16–18 weeks old NOD mice. Splenic mononuclear cells were isolated from female NOD mice at the indicated ages. The CD4^+^ T cells were purified by negative selection as described in Methods and stained with FITC-anti-CD4, APC-anti-CD25, and a PE-labeled I-A^g7^ tetramer loaded with the indicated peptide. The frequency of naïve CD4^+^CD25^-^PE^+^ T cells was determined by flow cytometry. The numbers of naive CD4^+^ T cells were calculated based on the total numbers of CD4^+^ T cells in each mouse. Data are expressed as the mean ± SD of each group (*n* = 8) of mice from at least three separate experiments. *** *p* < 0.001 for PPI_L4-20_ vs. insulin B_9-23_ by Student’s *t*-test.

We used I-A^g7^ tetramers presenting the PPI_L4-20_ to monitor the size of its naïve antigen-specific T cell pool during the development of unmanipulated NOD mice (Figure 2). We also used IA^g7^ tetramers to monitor the size of T cell pools recognizing the major target determinants within insulin B_9-23_ (InsBp8E [17,18,19,20]), and as a negative control, IA^g7^ tetramers presenting HEL_11-25_.

**Figure 3:**
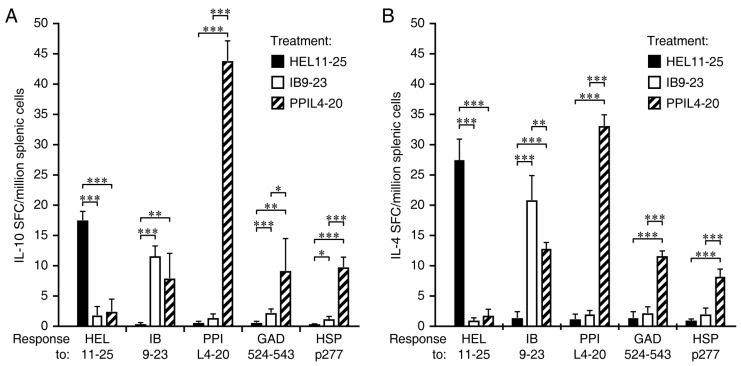
Immunization with PPI_L4-20_ induces robust IL-10 and IL-4 responses that spread to other ß-cell antigens in NOD mice. Female NOD mice at 15–16 weeks of age were immunized with HEL_11-25_ (black bars), insulin B_9-23_ (open bars), or PPI_L4-20_ (hatched bars) in alum IP on day 0 and 10, and ten days later (at about 18–19 weeks in age), their splenic mononuclear cells were tested for (**A**) IL-10 and (**B**) IL-4 responses to the antigens indicated on the X-axis by EPLISPOT assays. Data are expressed as the mean ± SD of SFC in each group (*n* = 5) from two separate experiments. The number of SFC in the control cells without antigen stimulation was ≤ 3, while the number of SFC in the positive controls with anti-CD3 stimulation was about 235. * *p* < 0.05, ** *p* < 0.01, and *** *p* < 0.001 by Student’s *t*-test. The frequency of naïve T cells reactive to these antigens in unvaccinated mice, as determined by tetramer staining, is shown in Figure 2.

The size of the HEL_11-25_ reactive naïve T cell pool increased slightly during NOD development (Figure 2) and comprised about 0.0025% of splenic CD4^+^ T cells at 16–18 weeks in age. The size of the naïve insulin B_9-23_-reactive T cell pool increased progressively from 2–14 weeks in age (peaking at about 0.008% of CD4^+^ T cells at 12–14 weeks in age) but then decreased greatly by 16 weeks in age, suggesting that cognate naïve T cells had been activated or recruited into the autoimmune response. The observed frequencies of naïve HEL_11-25_ and insulin B_9-23_-reactive splenic T cell pools are similar to those previously reported [28]. The proinsulin PPI_L4-20_ naïve T cell pool was similar in size and followed similar kinetics as that of naïve insulin B_9-23_-reactive T cells and increased from 2–14 weeks in age. However, while the size of the naïve insulin B_9-23_-reactive T cell pool declined precipitously after 12–14 weeks in age, the size of the PPI_L4-20_ naïve T cell pool remained stable thereafter (Figure 2). These observations are supported by the ELISPOT assessments following ASI, presented in Figure 3 below. Thus, analysis of L4-20-reactive naïve T cells indicates that there are large pools of naïve cognate T cells available for priming by ASI during the late stages of pre-T1D and after T1D onset.

### 3.3. Immunization with PPI_L4-20_, but Not Ins B_9-23_, Induces Robust IL-10 and IL-4 Responses Which Spread to Other ß-Cell Antigens

Fifteen-sixteen-week-old NOD mice were immunized IP twice with PPI_L4-20_, Ins B_9-23_, or HEL_11-25_ in alum and boosted with the same antigen after 10 days. Ten days later, at about 18–19 weeks in age, their splenic mononuclear cells were isolated and tested for the frequency of IL-10- and IL-4-secreting T cells responses to HEL_11-25_, insulin B_9-23_, PPI_L4-20_, GAD_524-543_, and HSP p277 by ELISPOT (Figure 3).

Immunization with the foreign antigen HEL_11-25_ in alum induced IL-10 and IL-4 T cell responses to the immunogen and these responses did not spread to any of the ß-cell autoantigens (black bars in Figure 3A,B, respectively), consistent with previous studies which have shown that administration of foreign antigens such as HEL or ß-galactosidase does not affect spontaneous T cell responses to ß-cell antigens or disease incidence in NOD mice (e.g., [9,24,29,30,31]). Immunization with insulin B_9-23_ induced moderate-low-level IL-10 and IL-4 responses to the immunogen at this late stage of pre-T1D, presumably because there were few naïve antigen reactive T cells left for priming by the immunization. Consequently, there was little or no spreading of IL-10 and IL-4 responses to other ß-cell antigens (open bars in Figure 3A,B, respectively) and no discernable spreading to PPI_L4-20_. In contrast, immunization with PPI_L4-20_ led to robust induction of IL-10 and IL-4 responses to PPI_L4-20_, which led to the strong spreading of IL-10 and IL-4 responses to other ß-cell antigens (hatched bars in Figure 3A,B, respectively).

### 3.4. Immunization Ins B_9-23_, but Not PPI_L4-20_, Primes IFNγ Responses and the Spreading of IFNγ Responses to Other ß-Cell Antigens

**Figure 4:**
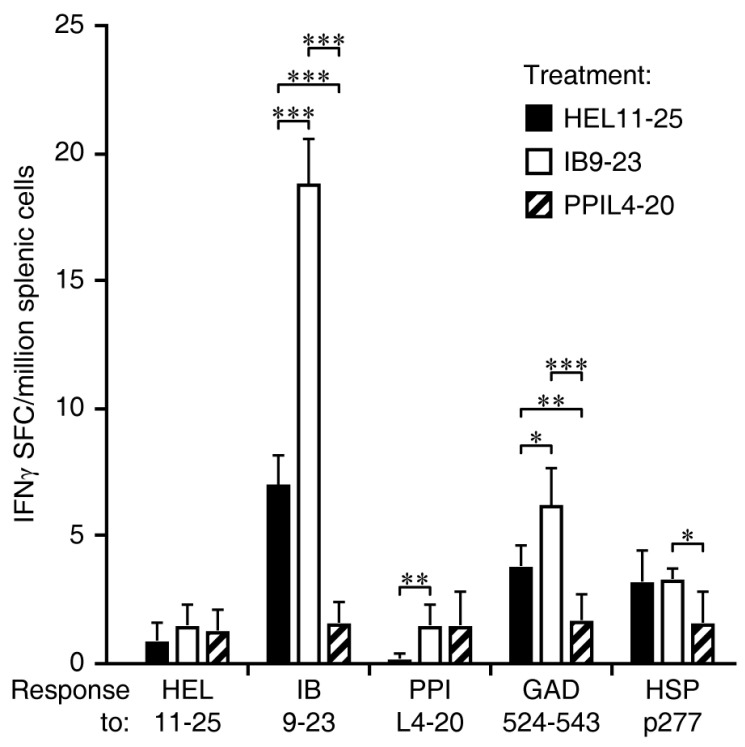
IFNγ-secreting T cell responses to a panel of ß-cell antigens following PPI_L4-20_ or insulin B_9-23_ immunization. Female NOD mice at 15–16 weeks of age were immunized IP with HEL_11-25_ (black bars), insulin B_9-23_ (open bars), or PPI_L4-20_ (hatched bars) in alum, boosted after 10 days, and ten days later (at about 18–19 weeks in age), their splenic mononuclear cells were isolated and tested for splenic IFNγ-secreting T cell responses to the antigens indicated on the X-axis by EPLISPOT assays. Data are expressed as the mean ± SD of SFC in each group (*n* = 5) from two separate experiments. The number of SFC in the control cells without antigen stimulation was ≤3 while the number of SFC in the positive controls with anti-CD3 stimulation was about 235. * *p* < 0.05, ** *p* < 0.01, and *** *p* < 0.001 by Student’s *t*-test.

In the same study, we studied how HEL_11-23_, insulin B_9-23_, and PPI_L4-20_ immunization affected IFNγ responses to ß-cell autoantigens (Figure 4). Immunization with the control foreign antigen HEL_11-25_ complexed with alum did not induce IFNγ responses to the immunogen, consistent with past studies showing that this adjuvant induces Th-2 biased immune responses in animals with no prior exposure to the immunogen [22]. As the 18–19 weeks old NOD mice that were studied here already have established autoimmune responses to insulin B_9-23_, GAD_524-543_, and HSP p277, we observed IFNγ responses to these antigens in HEL_11-25_-immunized mice, but no significant IFNγ responses were detected to PPI_L4-20_ consistent with it being an ignored determinant (Figure 4). Notably, immunization with insulin B_9-23_ greatly boosted IFNγ responses to insulin B_9-23_ (Figure 4). This led to the significant spreading of IFNγ responses to GAD_524-543_. Thus, immunization with an autoantigen determinant to which there are established autoimmune responses can both induce regulatory responses and boost pre-existing inflammatory responses that spread to other ß-cell antigens. In contrast, immunization with PPI_L4-20_ did not induce IFNγ responses to the immunogen or other ß-cell antigens (Figure 4). Rather, PPI_L4-20_ immunized mice had reduced IFNγ responses to insulin B_9-23_, GAD_524-543_, and HSP p277 relative to those in control HEL_11-25_-treated mice, indicating that the regulatory responses induced by PPI_L4-20_ immunization were able to reduce inflammatory responses to many ß-cell autoantigens.

### 3.5. L4-20 Effectively Induces Antigen-Specific CD4^+^ Treg and Tr1 Responses

**Figure 5:**
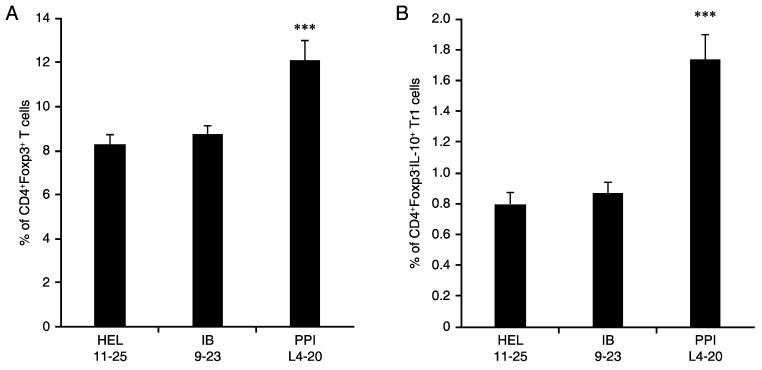
Immunization with PPI_L4-20_ increases the frequency of splenic Tregs and Tr1-like cells in NOD mice. Female NOD mice at 15–16 weeks of age were immunized IP with each antigen peptide in alum twice. Ten days later, their splenic mononuclear cells were isolated. The cells were stained with FITC-anti-CD4, fixed and permeabilized, followed by intracellular staining with PE-anti-Foxp3 or together with APC-anti-IL-10 for determining the frequency of (**A**) Tregs and (**B**) CD4^+^Foxp3^-^IL-10^+^ Tr1-like cells in total CD4^+^ T cells by flow cytometry. Data are expressed as the mean % ± SD of each group (*n* = 5) from three separate experiments. *** *p* < 0.001 vs. the other groups as determined by Student’s *t*-test.

NOD mice at 15–16 weeks in age were immunized IP with HEL_11-25_, insulin B_9-23_, or PPI_L4-20_ in alum, boosted after 10 days, and 10 days later, their splenic mononuclear cells were harvested and stained with FITC-anti-CD4 and APC-anti-IL-10 and PE-anti-Foxp3. FACS analysis showed that immunization with PPI_L4-20_ led to significantly increased frequencies of CD4^+^FoxP3^+^ Tregs (Figure 5A) and CD4^+^Foxp3^-^IL-10^+^ Tr1-like cells (Figure 5B) compared to immunization with HEL_11-25_ or insulin B_9-23_. These data demonstrate that immunization with an ignored ß-cell antigen can effectively induce antigen-specific CD4^+^ Treg and Tr1 responses at an advanced stage of pre-T1D.

### 3.6. Studies of Disease Reversal Using Intraperitoneal and Intradermal Routes of ASI Administration

Reversal of hyperglycemia in the NOD mouse model of T1D is considered to be the gold standard for preclinical evaluation of candidate interventive therapies. Since ASI monotherapies have not effectively reversed hyperglycemia in NOD mice after T1D onset (e.g., [1,25,32]), we combined PPI_L4-20_ or insulin B_9-23_ ASI with a suboptimal dose of oral GABA in order to functionally evaluate the ability of insulin B_9-23_ vs. PPI_L4-20_ to promote ß-cell tolerance. Our previous studies, as well as those of others, have shown that GABA treatment acts synergistically with ASI to (1) inhibit effector T cells, (2) enhance Treg responses, and (3) promote ß-cell health and replication [25,33,34,35,36,37]. Because combined treatment with GABA at an optimal dose (20 mg/mL) and an ASI is highly effective at reversing T1D in newly diabetic NOD mice [25], we administered GABA at a suboptimal low dose (6 mg/mL) in order to discern the ability of different ASI treatments to promote functional ß-cell tolerance. This low dose of GABA induced a brief remission in only about half of the treated diabetic mice which lasted less than two weeks [25]. We also assessed the effect of administering the ASI IP vs. intradermally.

NOD mice that displayed two consecutive daily blood glucose readings >250 but <300 mg/dL were considered newly diabetic and were randomized to receive PPI_L4-20_/alum or insulin B_9-23_/alum IP. Other groups of newly diabetic mice were given soluble PPI_L4-20_ or insulin B_9-23_ at a very low dose (1 μg in PBS) intradermally weekly for four weeks, as per [26]. The mice were provided GABA continuously through their drinking water after their first immunization. Disease remission was defined as two consecutive blood glucose readings <250 mg/dL, and thereafter, two consecutive blood glucose readings >250 mg/dL were considered as disease relapse.

**Figure 6:**
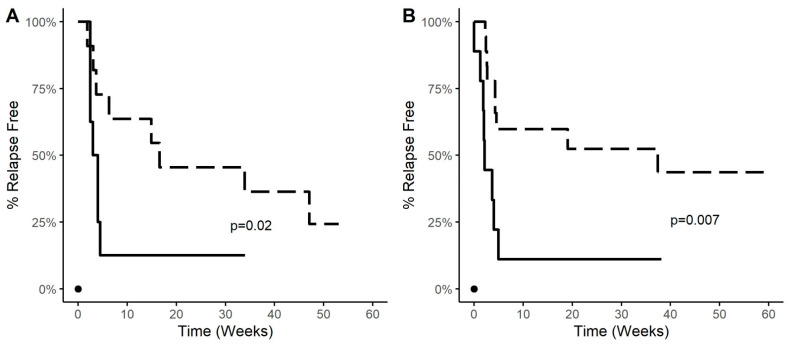
Percent relapse-free newly diabetic NOD mice given PPI_L4-20_ or insulin B_9-23_ intraperitoneally or intradermally together with oral low dose GABA. Newly diabetic NOD mice were given PPI_L4-20_ (dashed line) or insulin B_9-23_ (solid line) (**A**) intraperitoneally (with alum) or (**B**) intradermally (as soluble antigen) as described in Methods. All animals were then given low-dose GABA continuously through their drinking water and were monitored for disease recurrence. Data show the percentage of relapse-free mice in each mouse group following the treatment. In panel A, PPI_L4-20_ *n* = 11, and insulin B_9-23_ *n* = 8. In panel B, PPI_L4-20_ *n* = 18, and insulin B_9-23_ *n* = 9. *p* values were calculated by Log-Rank test. “●” indicates control untreated newly diabetic NOD mice (data from [25]). Previous studies of mice receiving low dose GABA (alone) observed that only about half of the treated mice went into remission and the responders relapsed within two weeks [25].

The longitudinal percentage of relapse-free mice in groups of mice treated with these peptides IP or intradermally in combination with oral GABA are shown in Figure 6A,B, respectively. Untreated newly diabetic NOD mice progressed to severe hyperglycemia without displaying disease remission (data shown is from our prior study [25]). As noted above, only about half of newly diabetic NOD mice respond to low-dose oral GABA monotherapy and these responders revert back to being hyperglycemic within two weeks [25]. In contrast, all of the mice given an ASI combined with oral GABA went into remission, aside from one mouse in the insulin B_9-23_ intradermal group. The majority of newly diabetic NOD mice treated with the major target determinant insulin B_9-23_/alum IP or with soluble insulin B_9-23_ intradermally, in combination with low-dose oral GABA, reverted to hyperglycemia within 5 weeks; however, about 15% remained disease-free for up to 32 weeks (Figure 6). In comparison, about 50% of the mice treated with PPI_L4-20_/alum IP or soluble PPI_L4-20_ intradermally combined with oral GABA remained normoglycemic for 30-weeks (Figure 6, *p* = 0.02 and 0.007 vs. insulin B_9-23_/alum IP and intradermal, respectively). There was no significant difference in time of disease remission between IP and intradermal PPI_L4-20_ treatments. Thus, after T1D onset, ASI using an ID determinant had a far greater therapeutic effect than an ASI based on a major target determinant.

## 4. Discussion

Immunological studies have shown that the frequency of cognate naïve T cell precursors determines the magnitude of the T cell response to an immunogen [7,8,9]. This strongly suggests that ASIs should utilize target cell antigens that have large pools of cognate naïve T cells available for priming toward regulatory responses. Identifying such antigens is complicated in the context of autoimmune disease because many autoantigen determinants become targeted by autoimmune responses leading to the activation of their cognate naïve T cells and the diminution of the naïve T cell pool that is available for priming by an ASI.

ASIs for T1D have focused on delivering whole autoantigens or peptides thereof containing major targets of the autoimmune response. As demonstrated herein and in our past studies, the ability of peptides containing target determinants to prime regulatory responses attenuates with disease progression [9,12]. Similarly, immunization with whole autoantigen induces responses primarily to the antigen’s dominant determinants, which to our knowledge, have always been the targets of the autoimmune response in NOD mice, and their ability to induce regulatory responses also attenuates with disease progression [9,12]. By using synthetic peptides from an autoantigen of interest, the natural processing of the whole antigen is circumvented, which can reveal very immunogenic autoantigen determinants that are not well presented from the whole antigen and had little impact on T cell selection. Both insulin B_9-23_ and PPI_L4-20_ had large pools of naïve precursor T cells (relative to HEL_11-25_) in young NOD mice, suggesting that their determinants may have had little impact on T cell selection—however, insulin B_9-23_-reactive T cells, but not PPI_L4-20_-reactive T cells, were drawn into the autoimmune response.

Evidently, following the processing of preproinsulin, the presentation of the determinant(s) within PPI_L4-20_ is insufficient to prime/activate their naive cognate T cells during the development of T1D in NOD mice. However, once experimentally activated by ASI, these T cells may respond to peptide/MHC that they had ignored as naïve cells. The immunogenicity of the antigen used for ASI appears to govern the extent to which ASI-induced regulatory responses spread intra- and inter-molecularly to other target cell antigens (Figure 3 and [9,29,30]). The observed spreading of IL-10 and IL-4 responses from PPI_L4-20_ to GAD_524-543_, insulin B_9-23_, and HSP p277 represents only a tiny fraction of the regulatory responses that are sure to have spread to many other ß-cell autoantigen determinants. Accordingly, the regulatory T cell responses arising secondarily from spreading vastly outnumber those induced to the immunogen itself (see also [9,38]). This spreading of anti-inflammatory responses may be largely due to the education of antigen-presenting cells toward anti-inflammatory phenotypes that then enhance the development of naive target determinant-reactive T cell precursors towards regulatory phenotypes upon their activation [39,40]. This may be the major factor behind PPII_L4-20′_s greater efficacy as an interventive therapy (when combined with GABA treatment) in newly diabetic NOD mice.

Notably, the PPI_L4-20_/alum-induced responses were robust and highly polarized toward regulatory responses, with no discernable induction of IFNγ responses. Moreover, PPI_L4-20_/alum treatment led to reduced IFNγ responses to insulin B_9-23_, GAD_524-543_, and HSP p277 (Figure 4). In contrast, while insulin B_9-23_/alum treatment moderately enhanced IL-10 and IL-4 responses to the immunogen, it also boosted IFNγ responses to the immunogen, GAD_524-543_, and presumably to other autoantigen determinants not tested here. These divergent outcomes may stem from the presence of activated and memory T cells responses to insulin B_9-23,_ but not to PPI_L4-20._ It appears that the increased presentation of insulin B_9-23_ following immunization promoted the proliferation of its activated and memory T cells. Accordingly, ID administration may be inherently safer than whole autoantigen or target determinant-based ASIs. Thus, even though PPI_L4-20_ and insulin B_9-23_ are derived from the same protein, their administration has very different outcomes based on the immunological history of the animal, as was previously demonstrated using other antigens [9,40].

In further regards to safety, our previous studies observed that ID-induced responses spread to ß-cell autoantigen target determinants but not to autoantigen ID determinants or to other ignored ß-cell antigens [9], which is consistent with observations in the current study. Accordingly, there does not appear to be a danger of ID-based ASIs causing autoimmune responses to spread to other non-targeted ß-cell antigens. It is also worth noting that while it is thought that ASI optimization needs information on which autoantigens are best suited for use at different stages of the autoimmune disease process, this issue may be bypassed by ID-based ASIs because the ID-reactive naïve T cell pool appears to be unaffected by the disease process.

Because ASI monotherapies have had little or no ability to reverse T1D after its onset in NOD mice, we combined ASI with a low dose of oral GABA, which has a rapid inhibitory action on autoreactive T cell responses and helps to preserve ß-cells while the protective adaptive immune response to the ASI slowly develop [25,34,35,41,42,43,44]. PPI_L4-20_ + GABA treatment effectively restored normoglycemia while insulin B_9-23_ + GABA had only a brief beneficial effect. These observations again associate the immunogenicity of the antigen used for ASI with the clinical outcome. We found that multiple low-doses of soluble PPI_L4-20_ intradermally were as effective as PPI_L4-20_/alum IP, which bodes well for the future development of the intradermal route of ASI administration in clinical applications for the treatment of autoimmune disease.

We have advocated for the use of autoantigen IDs in ASIs because their naïve cognate T cell pool does not appear to diminish with disease progression. While autoantigen IDs are straightforward to identify in the genetically similar NOD mice, their identification within the heterogeneous human population is more complex. One approach would be to utilize humanized mice that lack MHC I and II but express a human HLA, for example, HLA-DR4 or HLA-DQ8. These humanized mice would be immunized with peptides of a human autoantigen containing potential HLA-DR4 or HLA-DQ8 binding motifs but are not known to be targets of activated T cells in HLA-DR4 or HLA-DQ8-carrying individuals at risk for, or with, T1D. The peptides found to be immunogenic would be candidate IDs and further tested for their immunogenicity in healthy HLA-DR4 or HLA-DQ8 individuals and then those with established T1D, as measured by the induction of regulatory-type PBMC responses to the immunogen after vaccination. IDs with high immunogenicity would then be candidate ASIs for further clinical testing in HLA-DR4 or HLA-DQ8 individuals at risk or newly diagnosed with T1D.

Due to the complex nature of antigen processing and presentation by HLAs, it could be argued that there will not be commonly shared ID autoantigen determinants even among T1D patients carrying a particular HLA haplotype. Arguing against that scenario, it is notable that intralymphatic GAD65 with vitamin D supplementation slowed the loss of C-peptide production in new T1D patients carrying HLA DR3-DQ2, but not those negative for HLA DR3-DQ2 [5,6]. GAD65-immunized HLA DR3-DQ2 T1D patients also had greater GAD65-stimulated PBMC proliferation as well as IL-10 and IL-13 section compared with those negative for HLA DR3-DQ2 or patients in the placebo group [6]. These observations may stem from the particular GAD65 determinants presented by HLA DR3-DQ2 and the presence of large pools of naïve cognate T cells recognizing those determinants in these individuals. These findings suggest that in the diverse human population, there are HLA-associated commonalities in the processing and presentation of ASI antigens and that once HLA-associated IDs are discovered through the processes such as that described above, they may be generally applicable to individuals carrying those HLAs.

To our knowledge, no ASI clinical trial with T1D patients has examined the frequency of naïve T cell precursors in a patient’s PBMC that was capable of recognizing the ASI’s antigen prior to initiating the treatment. A number of studies have indicated that direct assessment of the frequency of human antigen-specific naïve T cells is feasible [8,45,46,47,48,49,50]. This may provide key information for helping to understand responsiveness vs. non-responsiveness to a particular ASI treatment.

Finally, it may be time to move beyond clinical trials that are fixed on testing a particular autoantigen as an ASI and move towards personalized ASI treatments. If HLA-associated autoantigen IDs are identified and become available for clinical use, ASIs could become agnostic in terms of the autoantigen to be delivered and utilize whichever autoantigen IDs have the most therapeutic potential in the context of different HLAs. An analysis of each patient’s PBMC to determine the frequency of naïve and activated T cells recognizing different autoantigen determinants could provide additional layers of information on which autoantigen peptides have high immunogenic potential and could be the most effective ASIs and which peptides may boost existing inflammatory T cell responses in each individual.

In summary, theoretical considerations concerning tolerance induction, advances in cancer immunotherapy using neoantigen vaccines, as well as our preclinical studies of targeted and ignored ß-cell autoantigen determinants in NOD mice, suggest that HLA-appropriate IDs of autoantigens may induce robust regulatory responses as ASIs for autoimmune disease with less likelihood of boosting inflammatory responses. These concepts provide a new framework for the selection of antigens to be used in personalized ASIs that are anticipated to have enhanced efficacy and safety for use as prophylactic monotherapies and in combined therapies for disease intervention.

## Data Availability

The raw data presented in this study are available on request from the corresponding author.
